# P-632. Safety of a Quadrivalent Meningococcal Conjugate Vaccine (MenACYW-TT) Administered Concomitantly with Routine Pediatric Vaccines in Healthy Infants and Toddlers

**DOI:** 10.1093/ofid/ofae631.829

**Published:** 2025-01-29

**Authors:** Abraham Moskow, Betzana Zambrano, Mandeep S Dhingra, Sandeep Gupta, Lucia Gan, Siham Bchir, Olga Syrkina, Audrey Hagenbach, Olga Lyabis, Christine Rehm

**Affiliations:** Rainbow Research, Inc, Barnwell, South Carolina; Sanofi Pasteur, Montevideo, Montevideo, Uruguay; Sanofi Pasteur, Montevideo, Montevideo, Uruguay; Sanofi, Swiftwater, Pennsylvania; Sanofi, Swiftwater, Pennsylvania; Sanofi, Swiftwater, Pennsylvania; Sanofi, Swiftwater, Pennsylvania; Sanofi, Swiftwater, Pennsylvania; Sanofi, Swiftwater, Pennsylvania; Sanofi, Swiftwater, Pennsylvania

## Abstract

**Background:**

MenACYW conjugate vaccine (MenACYW-TT) is a quadrivalent meningococcal conjugate vaccine, licensed (MenQuadfi^®^) for use in children ≥2 years of age in the US. In this modified double-blind, Phase-3 study (NCT03673462) conducted in the US and Puerto Rico, safety of MenACYW-TT vs a quadrivalent meningococcal vaccine MENVEO^®^ (MenACWY-CRM) was evaluated when co-administered with routine pediatric vaccines in healthy infants and toddlers.

**Figure:**
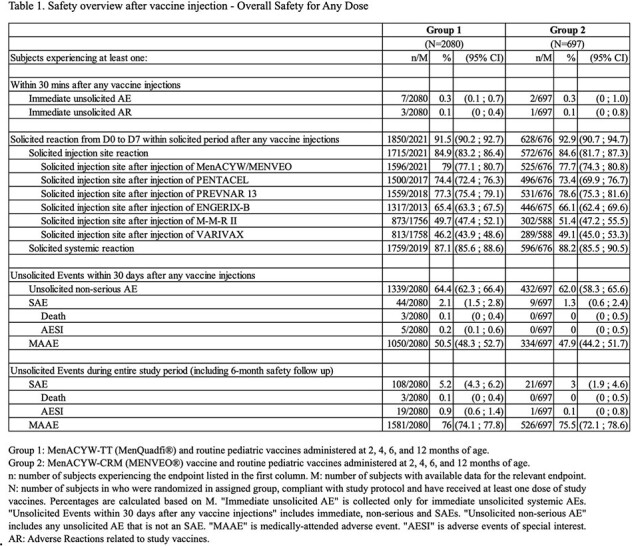

**Methods:**

Healthy infants and toddlers (age ≥ 42 to ≤ 89 days) were randomized 3:1 to receive either 4 doses of MenACYW-TT (Group 1) or 4 doses MenACWY-CRM (Group 2) at 2, 4, 6 and 12 months of age, and co-administered with routine pediatric vaccines following US schedules. Safety data collection included immediate unsolicited systemic adverse events (AEs) within 30 minutes post-vaccination, solicited AEs collected during the first 7 days of vaccination, serious adverse events (SAEs) including adverse events of special interest (AESIs), and medically-attended adverse events (MAAEs) collected throughout the study, including the 6-month follow up period.

**Results:**

A total of 2797 participants were randomized: 2099 to Group 1 and 698 to Group 2. Overall, 2777 participants received at least one (any) dose, and 2458 participants all 4 doses of the study vaccines. Within 30 minutes of any vaccination, 7 participants (0.3%) in Group 1 and 2 (0.3%) in Group 2 reported at least one immediate unsolicited AE. Within 7 days of any vaccination, participants who had at least 1 solicited injection site reaction in Group 1 and Group 2 were 79.0% and 77.7% respectively; 87.1% in Group 1 and 88.2% in Group 2 participants had at least one solicited systemic reaction. During the entire study, 5.2% in Group 1 and 3.0% in Group 2 participants experienced at least 1 SAE, with 0.9% in Group 1 and 0.1% in Group 2 who reported at least one AESI. Further, 1581 (76%) and 526 (75.5%) participants reported at least 1 MAAE. A total of 3 deaths were reported in group 1. All SAEs, AESIs and deaths were unrelated to the study vaccines (Table 1).

**Conclusion:**

The safety profiles of MenACYW-TT and MENACWY-CRM administered with routine pediatric vaccines were comparable in healthy infants and toddlers. No safety signals were identified.

**Disclosures:**

**Abraham Moskow, MD**, Sanofi: Investigator Meeting attendance **Betzana Zambrano, MD**, Sanofi: Employee|Sanofi: Stocks/Bonds (Public Company) **Mandeep S. Dhingra, MD**, Sanofi: Employee|Sanofi: Stocks/Bonds (Public Company) **Sandeep Gupta, MD**, Sanofi: Employee|Sanofi: Stocks/Bonds (Public Company) **Lucia Gan, PhD**, Sanofi: Employee|Sanofi: Stocks/Bonds (Public Company) **Siham Bchir, MSc**, Sanofi: Employee|Sanofi: Stocks/Bonds (Public Company) **Olga Syrkina, MD**, Sanofi: Employee|Sanofi: Stocks/Bonds (Public Company) **Audrey Hagenbach, n/a**, Sanofi: Employee|Sanofi: Stocks/Bonds (Public Company) **Olga Lyabis, MD**, Sanofi: Employee|Sanofi: Stocks/Bonds (Public Company) **Christine Rehm, MD**, Sanofi: Employee|Sanofi: Stocks/Bonds (Public Company)

